# Treatment Response, Survival Benefit and Safety Profile of PD-1 Inhibitor Plus Apatinib Versus Apatinib Monotherapy in Advanced Colorectal Cancer Patients

**DOI:** 10.3389/fonc.2022.863392

**Published:** 2022-05-19

**Authors:** Dengdeng Pan, Dongliang Liu, Lichuan Liang, Tongyi Shen, Chenzhang Shi, Huanlong Qin

**Affiliations:** ^1^Department of General Surgery, Anhui Medical University, Hefei, China; ^2^Department of Gastroenterology, Shanghai Tenth People’s Hospital, Tongji University School of Medicine, Shanghai, China; ^3^Department of General Surgery, Anhui Provincial Hospital Affiliated to the Anhui Medical University, Hefei, China

**Keywords:** PD-1 inhibitor plus apatinib, advanced colorectal cancer, treatment efficacy, survival outcome, safety profile

## Abstract

**Purpose:**

Programmed cell death protein 1 (PD-1) inhibitor plus apatinib is reported to be a promising strategy for advanced cancers. Moreover, a PD-1 inhibitor or apatinib exerts a certain efficacy in advanced colorectal cancer (CRC), whereas their synergistic effect is unclear. This study aimed to evaluate the treatment efficacy and safety of a PD-1 inhibitor plus apatinib in advanced CRC patients.

**Methods:**

In total, 45 advanced CRC patients who received a PD-1 inhibitor plus apatinib (PD-1 inhibitor plus apatinib group, N=20) or apatinib monotherapy (apatinib group, N=25) as third-line therapies were enrolled in the current study.

**Results:**

The objective response rate (20.0% vs. 8.0%) (*P*=0.383) and disease control rate (70.0% vs. 52.0%) (*P*=0.221) were numerically increased in the PD-1 inhibitor plus apatinib group, respectively, compared with the apatinib group, but no statistical significance was observed. The median progression-free survival (PFS) was 7.5 versus 4.8 months; the 1-year PFS rate was 32.5% versus 9.9%; the median overall survival (OS) was 12.3 versus 8.7 months; and the 1-year OS rate was 50.7% versus 27.0% in the PD-1 inhibitor plus apatinib group versus the apatinib group, respectively. PFS (*P*=0.038) and OS (*P*=0.048) were prolonged in the PD-1 inhibitor plus apatinib group compared with the apatinib group. PD-1 inhibitor plus apatinib (versus apatinib) was independently associated with longer PFS (*P*=0.012) and OS (*P*=0.009). The majority of the adverse events were of grade 1-2, wherein the incidence was similar between groups, except for the fact that the incidence of capillary proliferation was elevated in the PD-1 inhibitor plus apatinib group compared with the apatinib group (25.5% versus 0.0%) (*P*=0.013).

**Conclusion:**

PD-1 inhibitor plus apatinib presents a potential improvement in efficacy and survival benefit compared with apatinib monotherapy, with tolerable safety in advanced CRC patients.

## Introduction

Colorectal cancer (CRC) is a deadly cancer, with approximately 1.9 million cases of incidence and 0.9 annual million deaths (1, 2). Currently, advancements have been achieved in the surveillance and diagnosis of CRC *via* colonoscopy, computed tomography, or magnetic resonance imaging, which can help to identify CRC early (3, 4). Unfortunately, some CRC patients still develop metastatic disease at the time of diagnosis (5). Although systematic therapies, such as fluorouracil-based chemotherapy, cetuximab, bevacizumab, and other targeted therapies, as well as programmed cell death protein 1 (PD-1) inhibitors, have achieved survival benefits (to some extent) in advanced CRC patients, their 5-year survival rate is only 14% (5, 6). Therefore, more effective therapeutic approaches are needed for advanced CRC (7).

Apatinib is an innovative Chinese vascular epithelial growth factor receptor-2 inhibitor that suppresses tumor angiogenesis (8, 9). It has been reported that apatinib shows satisfactory efficacy in various advanced cancers, including CRC. For example, as a third-line therapy, apatinib has achieved a disease control rate (DCR) of 50.0% and a 1-year overall survival (OS) rate of 26.9% in metastatic CRC patients (10); in chemotherapy-refractory metastatic CRC, apatinib monotherapy showed satisfactory efficacy, with an objective response rate (ORR) of 8.3% and a DCR of 68.8%, with manageable toxicity (8). In addition, as a first-line therapy, apatinib plus leucovorin, 5-fluorouracil, and irinotecan (FOLFIRI) achieved a median OS of 16.1 months in metastatic CRC patients (11).

In addition to apatinib, the PD-1 inhibitor is another promising therapy for advanced cancer patients; this monoclonal antibody inhibits the binding between programmed death-ligand 1 (PD-L1) and PD-1, thereby promoting a T-cell-mediated anticancer effect (12). Recently, it has been proposed that PD-1 inhibitor-based combination therapy demonstrates promising therapeutic efficacy and tolerable safety in advanced cancer patients (13–15). In advanced CRC, a phase Ib trial demonstrated that a PD-1 inhibitor plus chemotherapy obtained a DCR of 43.3% (16); additionally, a PD-1 inhibitor showed favorable efficacy in patients with metastatic, microsatellite instability-high/mismatch repair-deficient (MSI-H/dMMR) CRC (17). Moreover, locally advanced CRC patients receiving a PD-1 inhibitor as neoadjuvant therapy showed a complete response (18).

The therapeutic efficacy of the combination of PD-1 inhibitors plus apatinib in patients with advanced cancer, including esophageal squamous cell carcinoma, gestational trophoblastic neoplasia, biliary tract cancer, and hepatocellular carcinoma, among other cancers, has been reported by previous studies (19–22). These studies have suggested that PD-1 inhibitors plus apatinib may be a promising strategy for advanced cancers. Due to the fact that PD-1 inhibitors and apatinib are both administered for advanced CRC, it can be deduced that PD-1 inhibitors plus apatinib may exhibit a synergistic effect in advanced CRC. However, few studies have investigated whether the combination of a PD-1 inhibitor and apatinib could further achieve benefit in advanced CRC. Therefore, the current study was designed to compare the treatment response, survival benefit, and safety profile of PD-1 inhibitor plus apatinib versus apatinib monotherapy as third-line therapies in advanced CRC patients.

## Methods

### Patients

In this prospective, observational cohort study, 45 patients with advanced CRC receiving a PD-1 inhibitor plus apatinib (n=20) or apatinib monotherapy (n=25) as third-line therapies from July 2019 to June 2021 were enrolled. The inclusion criteria were as follows: (1) clinicopathologic diagnosis of CRC; (2) age older than 18 years; (3) confirmed metastatic stage and inability to receive surgical resection; (4) failure or intolerance to previous second-line chemotherapy; (5) at least one measurable lesion, in terms of the Response Evaluation Criteria in Solid Tumors (RECIST, version 1.1); (6) Eastern Cooperative Oncology Group performance status (ECOG PS) 0 to 1; and (7) adequate hematologic, liver, and kidney function. The patients were ineligible for enrollment if they presented with one of the following conditions: (1) hypersensitivity to study drugs; (2) known contraindications to study drugs, such as gastrointestinal bleeding, uncontrolled hypertension, and grade 3-4 cardiac insufficiency; (3) other concomitant primary malignant diseases; and (4) pregnancy or being nursing mother. The Ethics Committee provided ethical permission for this study. Each patient provided a signed informed consent form.

### Treatment

Patients received PD-1 inhibitor plus apatinib (n=20) or apatinib monotherapy (n=25), depending on the disease condition and the decision made by the treating physicians. For these patients who received the PD-1 inhibitor plus apatinib, the alternative regimens were as follows: (1) intravenous administration of 200 mg of camrelizumab every three weeks and oral administration of 375 mg/day of apatinib, which could be adjusted to 250 mg/day, depending on the patient’s tolerance (23, 24); (2) intravenous administration of 200 mg of pembrolizumab every three weeks and oral administration of 375 mg/day apatinib, which could be adjusted to 250 mg/day, depending on the patient’s tolerance (25). Patients who chose to receive apatinib monotherapy received 500 mg of apatinib orally once daily, and the dosage of apatinib could be adjusted to 250 mg/day, depending on the patient’s tolerance (10). All of the patients continued the treatment until the occurrence of intolerable toxicity or disease progression.

### Follow-Up and Assessment

All of the patients were advised to return to the hospital for regular review and imaging examination, which was conducted to evaluate disease status every 4 to 8 weeks for the first three months and every two months thereafter until disease progression. The treatment response at month three was recorded for analysis, which was assessed according to RECIST, version 1.1. Moreover, the ORR and DCR were calculated. The last follow-up was completed in September 2021. The PFS and OS were imputed based on the follow-up records. The adverse events were recorded and graded according to the National Cancer Institute’s Common Terminology Criteria for Adverse Events (NCI-CTCAE), version 5.0, except cutaneous capillary proliferation, which is defined as hemangioma-like lesion according to a previous study (26).

### Statistical Analysis

SPSS 22.0 (IBM Corp., Armonk, New York, USA) was used for data processing and analyses. As appropriate, clinical characteristics between the two groups were examined *via* the t test, Wilcoxon rank-sum test, or χ^2^ test. The treatment response was compared between the two groups by using the χ^2^ test. The survival curves, including PFS and OS, were constructed by using the Kaplan–Meier method, and the variance analysis was determined *via* the log-rank test. The prognostic analysis was completed by using univariable and multivariable Cox proportional hazard regression methods. A *P*<0.05 indicated a statistically significant difference.

## Results

### Clinical Characteristics

In the PD-1 inhibitor plus apatinib and apatinib groups, the mean age was 57.2 ± 9.3 years and 55.3 ± 8.8 years, respectively. There were 7 (35.0%) female and 13 (65.0%) male patients in the PD-1 inhibitor plus apatinib group and 10 (40.0%) female and 15 (60.0%) male patients in the apatinib group. No difference between the groups was found regarding the clinical features, including age, sex, diagnosed tumor type, ECOG PS score, differentiation, number of metastatic sites, location of metastatic sites, KRAS mutation, or history of bevacizumab (all *P*>0.05) (**Table 1**).

**Table 1:** Clinical characteristics.

Items	Apatinib (N = 25)	PD-1 inhibitor plus apatinib (N = 20)	*P* value
Age (years), mean ± SD	57.2 ± 9.3	55.3 ± 8.8	0.487
Gender, No. (%)			0.731
Female	10 (40.0)	7 (35.0)	
Male	15 (60.0)	13 (65.0)	
Primary tumor site, No. (%)			0.731
Rectum	5 (20.0)	5 (25.0)	
Colon	20 (80.0)	15 (75.0)	
ECOG PS score, No. (%)			0.121
Score 0	8 (32.0)	11 (55.0)	
Score 1	17 (68.0)	9 (45.0)	
Differentiation, No. (%)			0.257
Well	3 (12.0)	2 (10.0)	
Moderate	13 (52.0)	7 (35.0)	
Poor	9 (36.0)	11 (55.0)	
Number of metastatic sites, No. (%)			0.289
Single	10 (40.0)	5 (25.0)	
Multiple	15 (60.0)	15 (75.0)	
Lung metastasis, No. (%)			0.182
No	15 (60.0)	8 (40.0)	
Yes	10 (40.0)	12 (60.0)	
Liver metastasis, No. (%)			0.419
No	6 (24.0)	7 (35.0)	
Yes	19 (76.0)	13 (65.0)	
Peritoneum metastasis, No. (%)			0.577
No	17 (68.0)	12 (60.0)	
Yes	8 (32.0)	8 (40.0)	
Other metastases, No. (%)			0.832
No	17 (68.0)	13 (65.0)	
Yes	8 (32.0)	7 (35.0)	
KRAS, No. (%)			0.380
Wild type	13 (52.0)	13 (65.0)	
Mutation	12 (48.0)	7 (35.0)	
History of bevacizumab, No. (%)			0.236
No	18 (72.0)	11 (55.0)	
Yes	7 (28.0)	9 (45.0)	
MSI-H		2 (10.0)	

PD-1, programmed cell death protein 1; SD, standard deviation; ECOG PS, Eastern Cooperative Oncology Group performance status; KRAS, Kirsten rat sarcoma 2 viral oncogene homolog; MSI-H, microsatellite instability-high.

### Comparison of Treatment Response

In the PD-1 inhibitor plus apatinib group, the numbers of patients who achieved CR, PR, SD, and PD were 0 (0.0%), 4 (20.0%), 10 (50.0%), and 4 (20.0%), respectively, whereas in the apatinib group, 0 (0.0%) patients achieved CR, 2 (8.0%) patients achieved PR, 11 (44.0%) patients had SD, and 9 (36.0%) patients received PD. The proportion of patients who achieved ORR (4 [20.0%] versus 2 [8.0%]) (*P*=0.383) and DCR (14 [70.0%] versus 13 [52.0%]) (*P*=0.221) in the PD-1 inhibitor plus apatinib group demonstrated an increasing trend over the apatinib group, whereas no statistical significance was observed (**Table 2**).

**Table 2:** Treatment response.

Items	Apatinib (N = 25)	PD-1 inhibitor plus apatinib (N = 20)	*P* value
Overall response, No. (%)			0.186
CR	0 (0.0)	0 (0.0)	
PR	2 (8.0)	4 (20.0)	
SD	11 (44.0)	10 (50.0)	
PD	9 (36.0)	4 (20.0)	
Not assessed	3 (12.0)	2 (10.0)	
ORR, No. (%)	2 (8.0)	4 (20.0)	0.383
DCR, No. (%)	13 (52.0)	14 (70.0)	0.221

PD-1, programmed cell death protein 1; CR, complete response; PR, partial response; SD, stable disease; PD, progressive disease; ORR, objective response rate; DCR, disease control rate.

### Comparison of PFS and OS

The median follow-up duration was 8.7 (range: 2.4-20.5) months, during which 37 (82.2%) patients suffered from disease progression, and 30 (66.7%) patients died.

**Figure 1:**
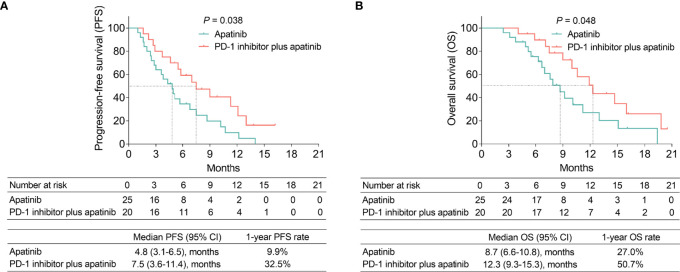
PFS and OS in the PD-1 inhibitor plus apatinib and apatinib groups. Comparison of PFS **(A)** and OS **(B)** between the groups.

The median PFS (95% confidence interval [CI]) in the PD-1 inhibitor plus apatinib group and the apatinib group were 7.5 (3.6-11.4) months and 4.8 (3.1-6.5) months, respectively; the 1-year PFS rates of these two groups were 32.5% and 9.9%, respectively. PFS was prolonged in the PD-1 inhibitor plus apatinib group compared with the apatinib group (*P*=0.038) (**Figure 1A**).

The median OS (95% CI) in the PD-1 inhibitor plus apatinib group and the apatinib group were 12.3 (9.3-15.3) months and 8.7 (6.6-10.8) months, respectively; the 1-year OS rates of these two groups were 50.7% and 27.0%, respectively. Moreover, OS was better in the PD-1 inhibitor plus apatinib group than in the apatinib group (*P*=0.048) (**Figure 1B**).

### Comparison of PFS and OS in Subgroups

In patients with a history of bevacizumab, OS was better in the PD-1 inhibitor plus apatinib group than in the apatinib group (*P*=0.018), whereas there was no difference in PFS (*P*=0.076) between these two groups (**Supplementary Figures 1A, B**); in patients without a history of bevacizumab, no difference was found in PFS (*P*=0.175) or OS (*P*=0.133) between these two groups (**Supplementary Figures 1C, D**).

In addition, in patients with a single metastatic site, OS was longer in the PD-1 inhibitor plus apatinib group than in the apatinib group (*P*=0.033); however, PFS was similar between these two groups (*P*=0.117) (**Supplementary Figures 1E, F**). In patients with multiple metastatic sites, no difference was found in PFS (*P*=0.241) or OS (*P*=0.549) between these two groups (**Supplementary Figures 1G, H**).

### Adjustment by Using Multivariable Cox Regression Analysis

The group (PD-1 inhibitor plus apatinib vs. apatinib) was associated with longer PFS (*P*=0.042, hazard ratio [HR]=0.499) but not with OS (*P*=0.053, HR=0.474) (**Supplementary Figures 2A, B**). After adjustments, group (PD-1 inhibitor plus apatinib vs. apatinib) was independently associated with longer PFS (*P*=0.012, HR=0.417) and OS (*P*=0.009, HR=0.348) (**Supplementary Figures 2C, D**). In addition, poor differentiation was also independently associated with worse DFS (*P*=0.011, HR=2.064) and OS (*P*=0.007, HR=2.423) (**Supplementary Figures 2C, D**).

### Comparison of Adverse Events

Most of the adverse events were similar between the groups, whereas only cutaneous capillary proliferation was increased in the PD-1 inhibitor plus apatinib group compared with the apatinib group (25.5% vs. 0.0%, respectively) (*P*=0.013). In the PD-1 inhibitor plus apatinib group, the primary grade 3 adverse events were neutropenia (10.0%), thrombocytopenia (10.0%), and anemia (5.0%); in the apatinib group, the significant grade 3 adverse events were hypertension (8.0%), anemia (4.0%), and neutropenia (4.0%). With these exceptions, no grade 4 adverse events were found in these two groups (**Table 3**).

**Table 3:** Adverse events.

Items	Apatinib (N = 25)					PD-1 inhibitor plus apatinib (N = 20)					*P* value^*^
	Total	Grade 1	Grade 2	Grade 3	Grade 4	Total	Grade 1	Grade 2	Grade 3	Grade 4	
Anemia, No. (%)	7 (28.0)	4 (16.0)	2 (8.0)	1 (4.0)	0 (0.0)	10 (50.0)	4 (20.0)	5 (25.0)	1 (5.0)	0 (0.0)	0.216
Fatigue, No. (%)	7 (28.0)	6 (24.0)	1 (4.0)	0 (0.0)	0 (0.0)	8 (40.0)	3 (15.0)	4 (20.0)	1 (5.0)	0 (0.0)	0.527
Neutropenia, No. (%)	6 (24.0)	3 (12.0)	2 (8.0)	1 (4.0)	0 (0.0)	8 (40.0)	4 (20.0)	2 (10.0)	2 (10.0)	0 (0.0)	0.336
Thrombocytopenia, No. (%)	7 (28.0)	5 (20.0)	1 (4.0)	1 (4.0)	0 (0.0)	7 (35.0)	3 (15.0)	2 (10.0)	2 (10.0)	0 (0.0)	0.749
Leukopenia, No. (%)	6 (24.0)	5 (20.0)	1 (4.0)	0 (0.0)	0 (0.0)	7 (35.0)	3 (15.0)	3 (15.0)	1 (5.0)	0 (0.0)	0.515
Proteinuria, No. (%)	6 (24.0)	5 (20.0)	1 (4.0)	0 (0.0)	0 (0.0)	7 (35.0)	3 (15.0)	3 (15.0)	1 (5.0)	0 (0.0)	0.515
Elevated transaminase, No. (%)	6 (24.0)	3 (12.0)	2 (8.0)	1 (4.0)	0 (0.0)	7 (35.0)	4 (20.0)	2 (10.0)	1 (5.0)	0 (0.0)	0.515
Hypertension, No. (%)	7 (28.0)	4 (16.0)	1 (4.0)	2 (8.0)	0 (0.0)	6 (30.0)	3 (15.0)	2 (10.0)	1 (5.0)	0 (0.0)	1.000
Hand-foot syndrome, No. (%)	7 (28.0)	4 (16.0)	2 (8.0)	1 (4.0)	0 (0.0)	6 (30.0)	3 (15.0)	3 (15.0)	0 (0.0)	0 (0.0)	1.000
Nausea and vomiting, No. (%)	6 (24.0)	3 (12.0)	2 (8.0)	1 (4.0)	0 (0.0)	6 (30.0)	4 (20.0)	2 (10.0)	0 (0.0)	0 (0.0)	0.741
Pruritus, No. (%)	5 (20.0)	4 (16.0)	1 (4.0)	0 (0.0)	0 (0.0)	6 (30.0)	4 (20.0)	2 (10.0)	0 (0.0)	0 (0.0)	0.500
Anorexia, No. (%)	4 (16.0)	3 (12.0)	1 (4.0)	0 (0.0)	0 (0.0)	5 (25.0)	2 (10.0)	3 (15.0)	0 (0.0)	0 (0.0)	0.482
Cutaneous capillary proliferation, No. (%)	0 (0.0)	0 (0.0)	0 (0.0)	0 (0.0)	0 (0.0)	5 (25.0)	3 (15.0)	2 (10.0)	0 (0.0)	0 (0.0)	0.013
Oral mucositis, No. (%)	3 (12.0)	2 (8.0)	1 (4.0)	0 (0.0)	0 (0.0)	4 (20.0)	3 (15.0)	1 (5.0)	0 (0.0)	0 (0.0)	0.682
Diarrhea, No. (%)	3 (12.0)	2 (8.0)	1 (4.0)	0 (0.0)	0 (0.0)	3 (15.0)	2 (10.0)	1 (5.0)	0 (0.0)	0 (0.0)	1.000
Creatinine elevation, No. (%)	2 (8.0)	2 (8.0)	0 (0.0)	0 (0.0)	0 (0.0)	3 (15.0)	2 (10.0)	1 (5.0)	0 (0.0)	0 (0.0)	0.642
Fever, No. (%)	2 (8.0)	1 (4.0)	1 (4.0)	0 (0.0)	0 (0.0)	3 (15.0)	2 (10.0)	1 (5.0)	0 (0.0)	0 (0.0)	0.642

*, P value was used to assess the difference of total adverse events incidence between groups.

PD-1, programmed cell death protein 1.

## Discussion

The treatment responses of apatinib or PD-1 inhibitor monotherapies in advanced CRC have already been reported by previous studies. However, the efficacy of PD-1 inhibitors plus apatinib in advanced CRC remains unclear. Our study compared the treatment response of CRC patients treated with a PD-1 inhibitor plus apatinib and apatinib monotherapy. The data showed that the proportion of patients achieving ORR (20.0% vs. 8.0%) and DCR (70.0% vs. 52.0%) was higher in the PD-1 inhibitor plus apatinib group than in the apatinib group, respectively, but the difference was not statistically significant. The possible reasons could be that (1) the binding between a PD-1 inhibitor and PD-L1 could promote antitumor immunity and inhibit immune escape, which may suppress CRC tumor growth (27); (2) apatinib may synergize the antitumor effects of a PD-1 inhibitor (28), thus indicating that PD-1 plus apatinib had potentially better treatment efficacy than apatinib monotherapy; and (3) the sample size of this study was relatively small, which resulted in low statistical power, which indicated no statistical significance was observed in the treatment response between groups.

Although several studies have proposed that PD-1 inhibitors or apatinib have a beneficial effect on the survival outcome of advanced CRC patients, the prognosis of advanced CRC is still far from satisfactory (8, 10, 29). Given that satisfactory survival rates have been achieved by PD-1 inhibitors plus apatinib in patients with advanced cancer (30, 31), it could be assumed that patients with advanced CRC may have better survival outcomes after treatment with PD-1 inhibitors plus apatinib. However, the relevant information is rare. In the current study, we observed that patients treated with PD-1 inhibitors plus apatinib had more satisfactory survival outcomes than those patients treated with apatinib monotherapy; moreover, PD-1 inhibitor plus apatinib was independently associated with longer PFS and OS. An explanation may be that patients who received a PD-1 inhibitor plus apatinib achieved a satisfactory treatment response, thus resulting in favorable survival outcomes. Another independent risk factor for the outcomes was poor differentiation, which is a well-recognized risk factor for a worsened prognosis in patients with CRC (32). Moreover, it was also observed that PD-1 inhibitor plus apatinib achieved better OS in patients with a history of bevacizumab. These data suggested that even after the failure of bevacizumab, PD-1 plus apatinib may also be effective. Furthermore, OS was higher after PD-1 inhibitor plus apatinib treatment than after apatinib monotherapy in patients with a single metastatic site, thus indicating that CRC patients with a single metastatic site could achieve greater benefit after PD-1 inhibitor plus apatinib treatment. However, these findings should be validated in a larger cohort.

The safety of PD-1 inhibitors plus apatinib in advanced CRC has not been reported. In contrast, in other cancers, it has been reported that the most common grade 3 or worse adverse events of PD-1 inhibitors plus apatinib include increased aspartate aminotransferase and increased gamma-glutamyl transferase in patients with advanced esophageal squamous cell carcinoma (19). Hypertension, rash, and neutropenia are the primary grade 3 treatment-related adverse events in patients with chemorefractory or relapsed gestational trophoblastic neoplasia treated with a PD-1 inhibitor plus apatinib (20). Regarding the safety of PD-1 inhibitors or apatinib monotherapies in advanced CRC patients, proceeding studies have suggested that pancreatitis, fatigue, and increased lipase levels are common adverse events with the use of a PD-1 inhibitor. At the same time, hypertension, hand-foot syndrome, and proteinuria are common adverse events with apatinib (17, 33). In our study, most of the adverse events were observed to be similar between the groups; in contrast, only the PD-1 inhibitor plus apatinib group had increased cutaneous capillary hyperplasia compared to the apatinib group, which could be explained by the fact that the administration of PD-1 inhibitor may lead to dysregulation of the immune system, thus resulting in cutaneous capillary proliferation (34). In addition, most of the adverse events were mild and manageable, thus suggesting that PD-1 inhibitor plus apatinib may be a safe option in advanced CRC patients.

Although many findings were identified in the current study, some limitations still existed. First, the sample size was relatively small, which may lead to low statistical power; therefore, the efficacy of PD-1 inhibitor plus apatinib as a third-line therapy in advanced CRC should be validated in a larger cohort. Second, some confounding factors may have affected the treatment response of the CRC patients in this study. For example, the previous use of the second-line chemotherapy may have affected the outcomes in the current study. Third, this was an observational, cohort study, which might induce potential bias; and the findings of this study should be verified in further randomized, controlled trials.

In conclusion, PD-1 inhibitor plus apatinib may have potential advantages over apatinib monotherapy in terms of treatment response and survival outcome in advanced CRC patients, and the safety profile is tolerable. The findings of the current study suggest that a PD-1 inhibitor plus apatinib may be a potential third-line regimen for advanced CRC. However, further studies should be conducted to provide more evidence for the recommendation of a PD-1 inhibitor plus apatinib as a third-line therapy in advanced CRC. Besides, the potential of PD-1 inhibitors plus apatinib as first- or second-line therapies for advanced CRC may be explored in the future. Moreover, the application of a PD-1 inhibitor plus apatinib as neoadjuvant therapy for CRC could be further explored.

## Data Availability Statement

The original contributions presented in the study are included in the article/**Supplementary Material**. Further inquiries can be directed to the corresponding authors.

## Ethics Statement

The studies involving human participants were reviewed and approved by Shanghai Tenth People’s Hospital, Tongji University School of Medicine. The patients/participants provided their written informed consent to participate in this study.

## Author Contributions

HQ and CS contributed to the conception. DP, TS, CS and HQ contributed to data acquisition and data analysis. DP, DL and LL drafted the manuscript. HQ and CS revised the manuscript. All authors read and approved the final manuscript.

## Funding

This study was supported by National Natural Science Foundation of China (No. 82070533).

## Conflict of Interest

The authors declare that the research was conducted in the absence of any commercial or financial relationships that could be construed as a potential conflict of interest.

## Publisher’s Note

All claims expressed in this article are solely those of the authors and do not necessarily represent those of their affiliated organizations, or those of the publisher, the editors and the reviewers. Any product that may be evaluated in this article, or claim that may be made by its manufacturer, is not guaranteed or endorsed by the publisher.

## Supplementary Material

Supplementary Figure 1 Subgroup analyses of survival outcomes. Differences in PFS **(A)** and OS **(B)** between the groups in CRC patients with a history of bevacizumab; differences in PFS **(C)** and OS **(D)** between the groups in CRC patients without a history of bevacizumab; differences in PFS **(E)** and OS **(F)** between the groups in CRC patients with a single metastatic site; differences in PFS **(G)** and OS **(H)** between the groups in CRC patients with multiple metastatic sites.

Supplementary Figure 2 Cox’s proportional hazards regression analysis for PFS and OS. Univariable Cox proportional hazards regression analysis for PFS **(A)** and OS **(B)** in CRC patients; multivariable Cox proportional hazards regression analysis for PFS **(C)** and OS **(D)** in CRC patients.
