# Supplementary figures and images for: Treatment Response, Survival Benefit and Safety Profile of PD-1 Inhibitor Plus Apatinib Versus Apatinib Monotherapy in Advanced Colorectal Cancer Patients
Source: Front Oncol. 2022 May 19;12:863392. doi: 10.3389/fonc.2022.863392 (PMC9160599; doi:10.3389/fonc.2022.863392)

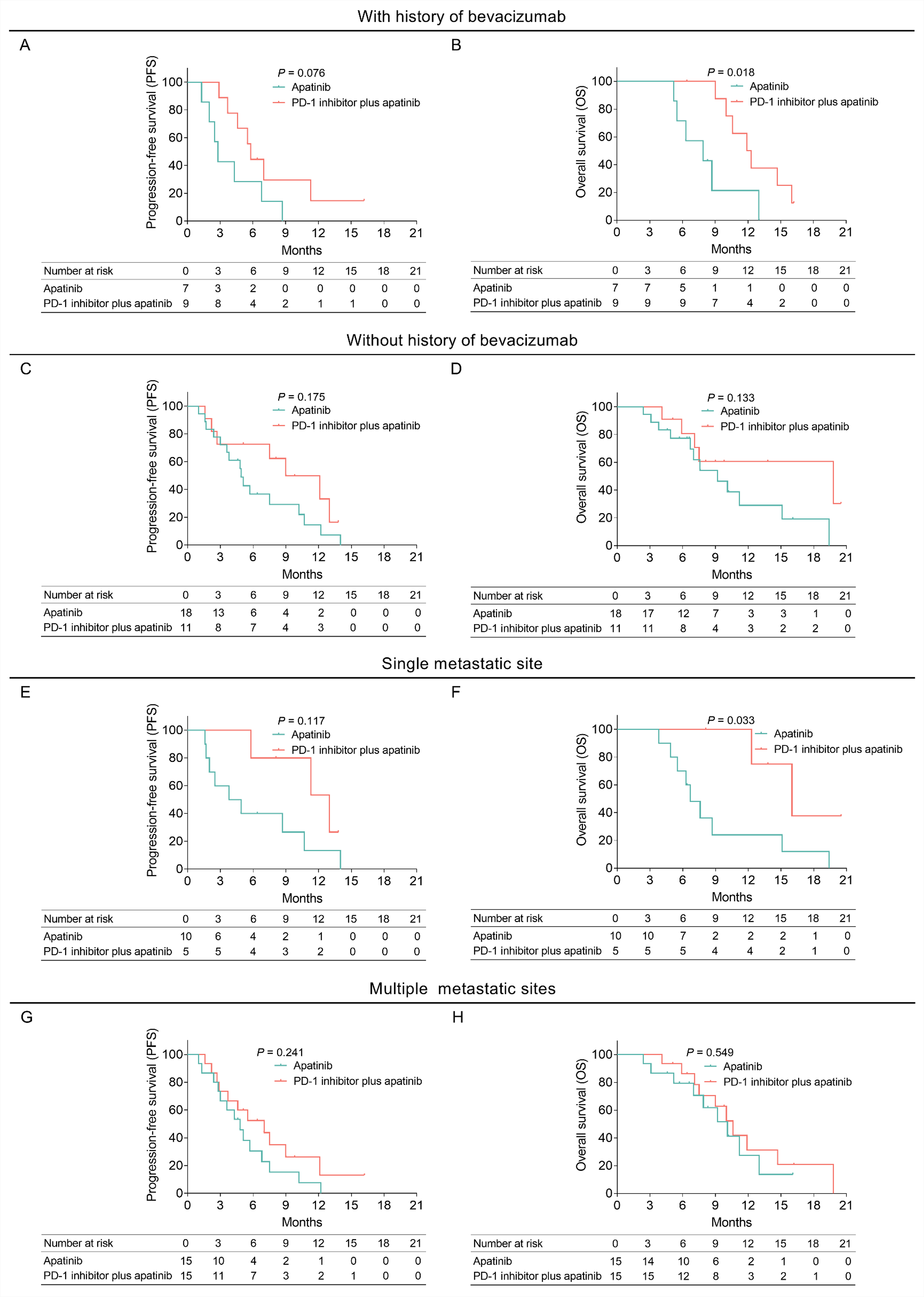

Supplement: Supplementary Figure 1 — Subgroup analyses of survival outcomes. Differences in PFS (A) and OS (B) between the groups in CRC patients with a history of bevacizumab; differences in PFS (C) and OS (D) between the groups in CRC patients without a history of bevacizumab; differences in PFS (E) and OS (F) between the groups in CRC patients with a single metastatic site; differences in PFS (G) and OS (H) between the groups in CRC patients with multiple metastatic sites. [file Image_1.tif]

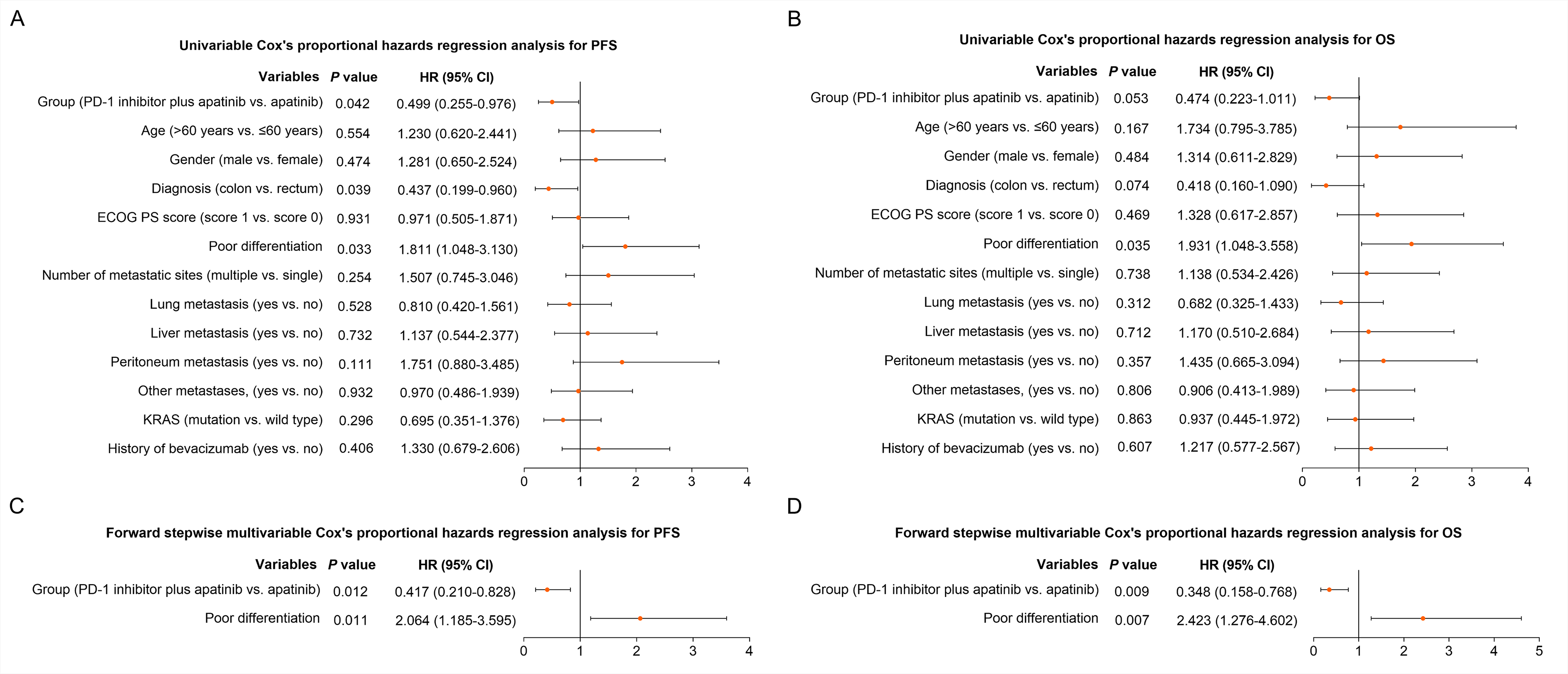

Supplement: Supplementary Figure 2 — Cox’s proportional hazards regression analysis for PFS and OS. Univariable Cox proportional hazards regression analysis for PFS (A) and OS (B) in CRC patients; multivariable Cox proportional hazards regression analysis for PFS (C) and OS (D) in CRC patients. [file Image_2.tif]
